# Effect of Ketamine on Post-Tonsillectomy Sedation and Pain Relief

**Published:** 2015-11

**Authors:** Seyed Alireza Bameshki, Mohammad Reza Salari, Mahdi Bakhshaee, Majid Razavi

**Affiliations:** 1 *Cardiac Anesthesia Research Center, Imam-Reza Hospital, Faculty of Medicine, Mashhad University of Medical Science, Mashhad, Iran.*; 2 *Sinus and Surgical Endoscopic Research Center, Ghaem Hospital, Faculty of Medicine, Mashhad University of Medical Sciences, Mashhad, Iran. *

**Keywords:** Children, Ketamine, Post-operative pain, Sedation, Tonsillectomy

## Abstract

**Introduction:**

Tonsillectomy is the one of the most common types of surgery in children, and is often accompanied by post-operative pain and discomfort. Methods of pain control such as use of non-steroidal anti-inflammatory drugs (NSAIDs), narcotics, and local anesthetics have been used, but each have their own particular side effects. In this study we investigated the effect of ketamine on post-operative sedation and pain relief.

**Materials and Methods:**

A total of 50 children aged between 5 and 12 years who were candidates for tonsillectomy were divided into two groups. The study group received ketamine-midazolam (ketamine 1 mg/kg, midazolam 0.1 mg/kg) and the control group received midazolam (0.1 mg/kg) in the pre-operative period. The same methods of anesthesia induction and maintenance were used in all patients. Pain score was assessed using the Wong-Baker Faces Pain Rating scale and sedation was evaluated using the Riker Sedation-Agitation scale at the time of extubation as well as 5, 10, 15, and 30 minutes and 1, 2, and 6 hours after surgery.

**Results:**

The two groups were similar in terms of age, weight, gender and duration of surgery. Pain after 15 and 30 minutes and agitation after 10 and 15 minutes following extubation were lower in the study group (ketamine-midazolam). Mean consumption and time of first request for analgesia after surgery as well as incidence of post-operative vomiting were similar in the two groups.

**Conclusion:**

Adding ketamine to midazolam in pre-operative of tonsillectomy reduces agitation and post-operative pain in the first 30 minutes after surgery.

## Introduction

Considering the high prevalence of infectious diseases of the pharynx, tonsils and adenoids in children, a high prevalence of tonsillectomy and adenotonsillectomy surgeries is also expected (1). Tonsillectomy is a common and painful procedure, often accompanied by complications such as laryngospasm, pain, bleeding, airway obstruction, nausea, vomiting, and aspiration (2). Since pain relief after tonsillectomy can help patients recommence food intake and can prevent patient dehydration secondary to low food intake, many efforts have been made to find an effective approach to pain management.

There are many methods available for reducing post-tonsillectomy pain, such as use of non-steroidal anti-inflammatory drugs (NSAIDs) (3), narcotics and infiltration of local anesthetics in tonsillar beds (4,5). Use of narcotics sometimes decreases upper airway tonicity and, with attenuation of cough reflex, causes respiratory depression and post-operative nausea and vomiting (3).

Ketamine has potent analgesic and sedative effects and, by inhibition of the N-methyl-D-aspartate receptors (NMDA) receptors in the spinal cord, reduces pain intensity and prevents it becoming chronic (6).

Despite the beneficial effects of ketamine in post-operative pain relief, there is some inconsistency in reported studies investigating the effects of intravenous ketamine (0.5mg/kg) on post-tonsillectomy pain (4,7-9). Thus, in this study, we investigated the effect of ketamine on post-tonsillectomy sedation and pain relief.

## Materials and Methods

This study was conducted following approval from the Ethics Committee of Mashhad University of Medical Sciences and after obtaining written informed consent from the parents of patients admitted to the teaching hospitals of Mashhad University of Medical Sciences. In total 50 patients aged between 5–12 years, rated as American Society of Anesthesiology (ASA) I–II, referred to the ear, nose, and throat (ENT) clinic of Imam Reza Educational Hospital, Mashhad, Iran who were candidates for tonsillectomy surgery, entered this prospective clinical trial. Patients were divided into two groups (study and control) using a random allocation table. All children were visited and examined the night before surgery. Patients were not allowed to eat solid food during the 8 hours before surgery and were not allowed to drink liquids during the 4 hours before surgery.

The primary outcome measure was evaluation of pain after tonsillectomy and the secondary outcomes were rate of sedation, nausea, and vomiting. Variables included weight, age, gender, pain intensity, time to first analgesic prescription, sedation, dose of pethidine, and incidence of vomiting and duration of surgery. Exclusion criteria were history of lung disease and motion sickness, prolonged surgery (more than 2 hours), and bleeding greater than 10cc/kg.

After the child reclined on the bed, standard monitoring including pulse oximetry, electrocardiogram, and non-invasive blood pressure measurement was started. Two syringes were prepared with the same volume of solution. One syringe contained midazolam 1mg/ml (control group) and the other syringe contained midazolam 1mg/ml with ketamine 10mg/ ml (study group). Both syringes were clear and colorless, and the anesthesiologist was not aware of the content of the syringes. Two minutes before induction of anesthesia, 0.1mg/kg of the contents of the syringe was injected into the patients. Anesthesia induction in children was accomplished using fentanyl 2µg/kg, thiopental Na 5 mg/kg, and atracurium 0.6 mg/kg, then tracheal intubation was performed using a tracheal tube, and intravenous (iv) dexamethasone 0.1mg/kg was given to all patients. Anesthesia maintenance was managed with O_2 _and N_2_O, with proportions of 30% and 70%, respectively, and propofol 100–150µg/ kg/h was infused. During surgery, crystalloid was administered according to the 4:2:1 rule.

At the end of surgery, the mouth and throat of patients was irrigated with normal saline and patients were transferred to the recovery room. After the return of spontaneous breathing with adequate volume and airway reflexes, the endotracheal tube was removed and patients were placed in the lateral position with the head down and received 6 L/min O_2_ via a face mask. Patients in the recovery room at time of extubation, and 5,10,15, and 30 minutes and 1,2, and 6 hours afterwards were evaluated for pain. Time and dose of prescribed analgesic were also recorded. The Wong-Baker Faces Pain Rating scale (0=no pain; 5= worst pain) was used for assessment of post-operative pain and the Riker Sedation -Agitation scale (SAS; 1= unarousable; 7=dangerous agitation) was used for assessment of the patients’ level of sedation (10,11).

The prevalence of nausea in children was not recorded because of difficulties in definition and recording; however, post-operative vomiting was recorded. In the case of more than one episode of vomiting, iv metoclopramide15 mg/kg was administered.

In case of the need for analgesia (Faces score ≥4), pethidine was injected (iv, 0.5 mg/kg) and the time of injection was recorded. During first 24 hours of admission after surgery, the patients’ pain was controlled using this method. Indices of discharge from the recovery room to the ward were full consciousness, normal vital signs, proper pain control and absence of nausea and vomiting. 

Quantitative data were compared between the two groups using either Student’s t-test or the Mann-Whitney test. For qualitative data, the Chi-square test and Fisher’s exact test were applied. SPSS software version 11 was used for data analyses and P<0.05 was considered statistically significant.

## Results

Assessment of patients’ demographic characteristics showed a mean age of 6.5 ± 1.94 years and a mean weight of 21.10 ± 5.30 kg. Thirty-four patients were male (64%) and 16 were female (32%). There was a normal distribution between the two groups in terms of age, weight and gender (P=0.720, P=0.109,P=0.225. respectively).

Duration of surgery in the midazolam group was 84/20±32/49 minutes, compared with 71/00± 32/60 minutes in the ketamine-midazolam group, and in general was 77.60±32.89. There was no difference between the two groups (P=0.158). 

**Fig 1:**
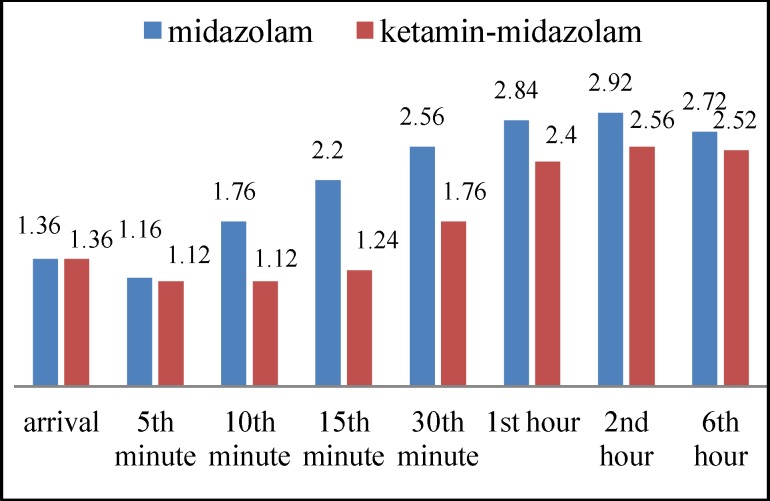
Comparison of pain between study groups

In the recovery room there was no significant difference between the two groups with respect to pain score at time of extubation, or after 5 or 10 minutes (P=0.007, P= 0.47), but it was significantly higher in the midazolam group at 15 and 30 minutes after extubation; indicating that patients in this group endured more pain (P=0.007, P=0.47) (P=0.058, P=0.890, P=1, respectively). In the ward and 1, 2, 6 hours after surgery, there was no significant difference between the two groups with respect to pain score (P=0.385, P=0.137, P=0.255, respectively) (Fig.1).

**Fig 2:**
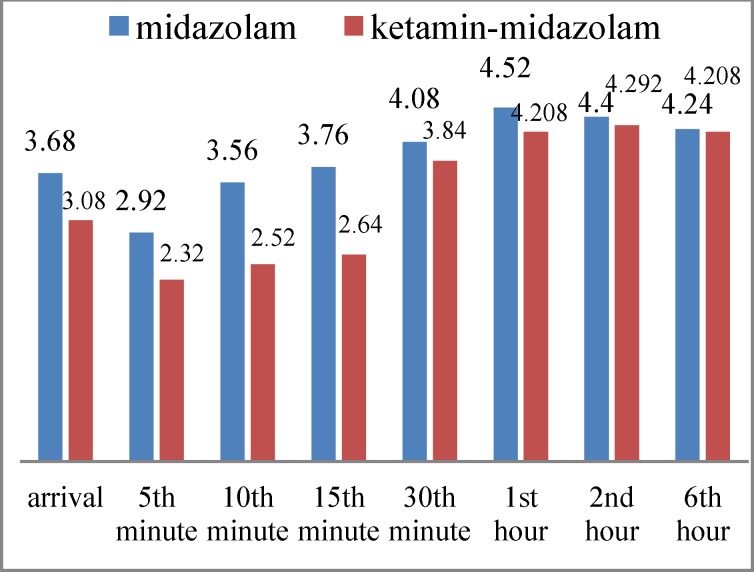
Mean sedation rate in study groups

There was no significant difference between the two groups with respect to sedation score based on the SAS scale at time of extubation, 5 and 30 minutes and 1,2 and, 6 hours later, but it was significantly higher in the midazolam group at 10 and 15 minutes after extubation; indicating that patients were more agitated in this group (P=0.006, P=0.002) (Fig.2).

The average consumption of analgesia after surgery and in the first day of admission was similar in the two groups, but time to request for analgesia was longer in the ketamine-midazolam group, although the difference was not statistically significant (P=0.276, P=0.166). 

Five patients experienced post-operative vomiting (10%); one (4%) in the midazolam group and four (16%) in the ketamine-midazolam group. There was no significant difference between the two groups in rate of post-operative vomiting (P=0.157).

## Discussion

Several studies have investigated different methods of post-tonsillectomy pain control and sedation. Comparisons between narcotics and other drugs for sedation and pain relief have been performed in numerous studies. Engelhardt et al. examined iv morphine and tramadol in pain relief and showed that, despite more side effects with morphine, pain relief for the two drugs were similar (12). A similar comparison in the study by Ozalevi et al. also suggested that tramadol is an appropriate substitute with lower side effects for post-tonsillectomy pain control in children (13). In addition, a study by Chew confirmed that tramadol had a greater analgesic effect and that patients had improved consciousness after surgery (14). Viitanen et al. compared tramadol with ibuprofen, and the results showed that despite similar duration of recovery and side effects, tramadol had a greater post-operative analgesic effect (15). Pendville et al. compared tramadol with paracetamol, and reported that tramadol had a greater sedation effect than paracetamol, despite similar side effects (16). Our study compared midazolam with ketamine-midazolam in terms of pain relief and agitation and showed that ketamine, like tramadol, could achieve greater pain relief and less agitation. In 2008, Ugur et al. compared intramuscular and peritonsillar infiltration of tramadol in children undergoing tonsillectomy and showed greater pain relief in peritonsillar infiltration of tramadol (17). These results were confirmed in a study by Atef et al. which used an objective pain scale (18). 

In all the studies mentioned above, the performance of either local, systemic or intramuscular tramadol was associated with decreased tonsillectomy pain compared with morphine and paracetamol. The common mechanism of inhibition of the NMDA receptor suggests that ketamine in place of tramadol would avoid the side effects of narcotics and tramadol.

The Umuroglu et al. study compared ketamine, morphine and tramadol in this matter. Pain in children was assessed using the Children’s Hospital of Eastern Ontario Pain Scale (CHEOPS) and numeric rating scale (NRS), and morphine was introduced as the best analgesic for post-tonsillectomy pain in children (4). Our study confirmed the improved analgesic effect of ketamine-midazolam compared with midazolam. The Umuroglu study also confirmed the analgesic effect of ketamine, but the effect was less than that of morphine.

A study reported by Erhan in which 0.5 mg/kg ketamine (similar dose to Umuroglu study) was used and the CHEOPS and Wilson sedation scales were assessed showed that ketamine reduces pain and need for analgesia and increases the time to first request for analgesia, without significant changes in heart rate or nausea and vomiting (8). The similar Murray study showed that 0.5 mg/kg iv ketamine reduces pain without significant increase in rates of sedation and hallucination (9). The results of these studies are consistent with the findings of our study.

A study reported by Elhakim investigated the effect of intramuscular ketamine, and the results showed pre-operative intramuscular (IM) ketamine reduces post-operative pain and odynophagia and also affects analgesic dose and time to first use of analgesia (19). Our study confirmed the effectiveness of ketamine on pain relief.

However, unlike the above studies, a study reported by Batra with 40 patients who received anesthesia with 0.5 mg/kg ketamine and remifentanil maintenance showed no effect of ketamine on pain relief (as evaluated using CHEOPS and a visual analog scale [VAS]) and suggested that a low dose of ketamine does not reduce post-operative pain in surgery with remifentanil anesthesia maintenance (20). Also, the study by Abu-Shahwan demonstrated the ineffectiveness of adding 0.25 mg/kg ketamine to morphine in the pain relief of children undergoing tonsillectomy (evaluated using CHEOPS) (21). These results are inconsistent with ours, although the method of adding ketamine to morphine or different methods of anesthesia maintenance could be responsible for this difference. Time of prescription of ketamine was investigated in the study by Da Conceição, in which patients were assigned into three groups (controls, ketamine before surgery, and ketamine post-tonsillectomy [0.5mg/kg]). Pain was evaluated using the Oucher scale and the results showed that ketamine significantly reduces post-operative pain (22). Although this study used a different method of pain assessment, as with our study findings, it showed that the perioperative admin- istration of ketamine could reduce post-operative pain.

In our study, there was less pain at 15 and 30 minutes and less agitation at 10 and 15 minutes after extubation with ketamine-midazolam injection compared with midazolam. However, average pain and sedation were lower in the ketamine group at all times, although the difference only reached statistical significance at the times stated above. The average analgesic consumption and time of first request for analgesia post-operation and the incidence of nausea and vomiting were similar in the two groups. The remaining effects of ketamine analgesia could explain reduced pain and agitation in the first half hour after surgery and the lack of a significant difference after the ketamine effect had subsided.

## Conclusion

Adding ketamine to midazolam premedi- cation in tonsillectomy reduces post-operative pain and agitation in the first half hour after surgery.
